# Self-Awareness of Psychopathology and Brain Volume in Patients With First Episode Psychosis

**DOI:** 10.3389/fpsyt.2019.00839

**Published:** 2019-11-15

**Authors:** Jeong-Youn Kim, Hyeonjin Jeon, Aeran Kwon, Min Jin Jin, Seung-Hwan Lee, Young-Chul Chung

**Affiliations:** ^1^Clinical Emotion and Cognition Research Laboratory, Inje University, Goyang, South Korea; ^2^Department of Psychology, Chung-Ang University, Seoul, South Korea; ^3^Department of Psychiatry, Inje University, Ilsan-Paik Hospital, Goyang, South Korea; ^4^Department of Psychiatry, Chonbuk National University Medical School, Jeonju, South Korea

**Keywords:** schizophrenia, prospective and retrospective memory questionnaire, ruminative response scale, interpersonal sensitivity measure, right superior temporal gyrus

## Abstract

Memory impairment, excessive rumination, and increased interpersonal sensitivity are major characteristics of high psychosis risk or first episode psychosis (FEP). Herein, we investigated the relationship between brain volume and self-awareness of psychopathology in patients with FEP. All participants (FEP: 34 and HCs: 34) completed clinical assessments and the following self-reported psychopathology evaluations: prospective and retrospective memory questionnaire (PRMQ), ruminative response scale (RRS), and interpersonal sensitivity measure (IPSM). Structural magnetic resonance imaging was then conducted. The PRMQ, RRS, and IPSM scores were significantly higher in the FEP group than in the healthy controls (HCs). The volumes of the amygdala, hippocampus, and superior temporal gyrus (STG) were significantly lower in the FEP group than in the HCs. There was a significant group-dependent moderation effect between self-awareness of psychopathology (PRMQ, RRS, and IPSM scores) and right STG (rSTG) volume. In the FEP group, self-awareness of psychopathology was positively associated with rSTG volume, while in the HCs, this correlation was negative. Our results indicate that self-awareness of psychopathology impacts rSTG volume in the opposite direction between patients with FEP and HCs. In patients with FEP, awareness of impairment may induce increases in rSTG brain volume. However, HCs showed decreased rSTG volume when they were aware of impairment.

## Introduction

First episode psychosis (FEP) is defined as the first time a person outwardly shows symptoms of psychosis. When patients with FEP become aware of their problems, they show distress and confusion, ruminate their symptoms, and have interpersonal problems caused by enhanced sensitivity (1). Conversely, after experiencing chronic overt psychotic symptoms, patients with chronic schizophrenia generally show poor insight into their own symptoms (2).

Self-awareness of psychopathology is one dimension of clinical insight (3). In psychiatric patients, lack of self-awareness of illness is associated with poor psychosocial function (4), poor clinical outcomes (5), and poorer treatment adherence (6), while in healthy controls, greater self-awareness of illness may lead them to adopt the identity of a “psychiatric patient” to themselves (7), which may be associated with poorer social functioning (8) and lower self-esteem (9). Previous studies have found that patients with full-blown schizophrenia lack self-awareness of illness (4, 10). About 46% of FEP patients showed poor insight (11) and insight impairment is associated with multiple cognitive deficits (12).

The subjective evaluation of self-awareness of illness can also be important in the early detection of schizophrenia, because complaints precede prodromal symptoms and are therefore useful in predicting onset and relapse of schizophrenia (13, 14) as well as long-term symptomatic deterioration (15). Subjective measures of cognitive function, such as self-report questionnaires or related scales, shed light on self-perceived cognitive difficulties that occur during daily activity and cannot be observed using behavioral tests (16). Although some self-reported scales measure clinical insight, such as the schedule of assessment of insight-expanded version (SAI-E) (17 1997) and the Beck cognitive insight scale (BCIS) (18), they are too broad to examine self-awareness of illness among patients with FEP. In particular, the SAI-E measures overall relabeling of symptoms, awareness of illness, and need for treatment, while the BCIS measures self-certainty and self-reflectiveness. Therefore, self-awareness of illness should be measured using other self-reporting scales that measure specific cognitive functions related to the pathological problems seen in patients with FEP.

In addition to cognitive deficits, patients with FEP seem to display memory problems, rumination, and interpersonal sensitivity (19–22). Bigdeli et al. (21) revealed that patients with FEP performed more poorly than a healthy group in a self-reporting memory task. In contrast, patients with schizophrenia did not report subjective complaints significantly more often than controls (16).

Rumination is described as a cognitive process that includes repetitive, prolonged, and recurrent thoughts about oneself, one’s concerns, and one’s experiences (23). Rumination is related to hallucination-proneness and to a range of mild abnormal experiences, including unrealistic feelings, perceptual alterations, and temporal disintegration (24, 25), as well as to negative symptoms such as stereotyped thinking and emotional withdrawal (26). One experiment revealed that antecedent rumination and worry predicted persecutory delusions and auditory hallucinations; it also predicted the degree of anguish associated with these psychotic experiences in young adults with psychosis (22). In fact, rumination may be the reason some young people who experience psychotic symptoms become distressed and seek help, while others do not (27, 28).

To understand psychosis, one important aspect of interpersonal interactions is interpersonal sensitivity, which is a personality trait described as excessive awareness of the behavior and feelings of others (29). Interpersonal sensitivity is associated with the risk of paranoid thinking in the general population (30). Early studies indicated that high interpersonal sensitivity occurs among other subjective symptoms and observable behavioral changes during the prodromal phase of schizophrenia (19, 30).

With regards to the brain abnormalities related to psychosis, one meta-analysis found that gray matter decreases were common in the thalamus, the left uncus/amygdala region, the insula bilaterally, and the anterior cingulate in patients with both first episode psychosis (FEP) and chronic schizophrenia (31). Furthermore, the decreases in gray matter volume in the bilateral caudate head and right bilateral superior parietal lobule were more severe in first-episode or early stage schizophrenia than in chronic schizophrenia (32, 31). Another study found that the volumes of the frontal and temporal areas were decreased in first-episode schizophrenia, but less than in chronic schizophrenia (33). The superior frontal gyrus has been associated with anomalies in self-awareness, social cognition, and emotion (34, 35), while the superior temporal gyrus (STG) and subcortical regions such as the thalamus are associated with positive symptoms, including auditory hallucinations, thought disorders (36, 37), deficits in working memory and attention (38, 39), and social processing (40–42).

In the present study, we explored the relationship between brain volume and self-awareness of psychopathology in FEP. We hypothesized that patients with FEP show greater self-reported memory impairment, rumination, and interpersonal sensitivity than healthy participants, and that these abnormalities are associated with brain morphological anomalies that are vulnerable targets of FEP.

## Materials and Methods

### Participants

A total of 34 patients [male: 16, female: 18, age: 28.353 ± 7.261 (range: 19–47)] who met the Diagnostic and Statistical Manual of Mental Disorders, fifth edition (DSM-V) criteria for first-episode schizophrenia, schizophreniform disorder, schizoaffective disorder, or psychotic disorder not otherwise specified (NOS), as assessed using the Structured Clinical Interview for DSM-V Axis I disorders (43) (SCID-I), were eligible to participate in the study. Patients were included if they had symptoms requiring antipsychotic treatment [a score of ≥4 (moderate) on at least one of the following: the PANSS (44) positive items, which were P1, P2, P3, P5, and P6, or Clinical Global Impression (CGI) score ≥4] with illness duration of more than 1 month and less than 60 months (45), and no lifetime history of previous antipsychotic exposure lasting for 2 or more consecutive weeks (46).

A total of 34 healthy control (HC) participants [male: 21, female: 13, age: 31.647 ± 6.764 (range: 21–47)] were recruited from the local community *via* newspapers and flyers. An initial screening interview was used to exclude subjects with any identifiable neurological disorder, head injury, or any personal or family history of psychiatric illness. After the initial screening, potential HCs were interviewed using the Structured Clinical Interview for DSM V for Axis I Psychiatric Disorders (43) and were excluded if they had any such disorders. All participants were right-handed according to the Edinburgh Handedness Inventory (47). All procedures followed were in accordance with the ethical standards of the Ethics Committee of Chonbuk National University Hospital, Republic of Korea (approval number: CUH2014-11-002) and with the Declaration of Helsinki. All participants provided written informed consent themselves or from their legal guardians if they were the minors.

### Psychological Measures

#### Self-Awareness of Psychopathology: Subjective Symptoms

Self-awareness of psychopathology was assessed using self-report measures, namely, the prospective and retrospective memory questionnaire (PRMQ), the ruminative response scale (RRS), and the interpersonal sensitivity measure (IPSM).

The PRMQ (48, 49) was used to measure subjective complaints of memory impairment. It is a 16-item questionnaire. Each participant was asked to rate how often each type of memory failure occurred in their daily life on a 5-point scale (very often, quite often, sometimes, rarely, and never). The PRMQ comprises six subscales: prospective, retrospective, short-term, long-term, self-cued, and environmentally-cued. Of the 16 PRMQ items, eight inquire about prospective memory and eight inquire about retrospective memory. The questionnaire also contains an equal number of items concerned with self-cued memory and environmentally-cued memory, and with short-term and long-term memory. Thus, each PRMQ item can be categorized along three dimensions. For example, “If you tried to contact a friend or relative who was out, would you forget to try again later?” is categorized as measuring prospective, long-term, self-cued memory. Thus, the PRMQ may have an advantage over other self-report scales in that it balances prospective with retrospective items and measures these constructs systematically over a range of contexts.

The RRS (50, 51) comprises three subscales: depression-related, reflection, and brooding. It consists of 22 items, each of which is rated using a 4-point Likert scale, ranging from 1 (“almost never”) to 4 (“almost always”). Among the 22 RRS items, 12 are concerned with depressive symptoms (e.g., “Think about how alone you feel”), five inquire about brooding (e.g., “Think about a recent situation, wishing it had gone better”), and five address reflective pondering or reflection (e.g., “Analyze recent events to try to understand why you are depressed”) (51).

The IPSM has been used to assess excessive sensitivity to the interpersonal behavior of others, to social feedback, and to negative evaluation by others (29). The original IPSM scale comprises 36 items. However, we used the validated Korean version of the IPSM, which consists of 24 items (52) rated using 4-point Likert scale ranging from 1 (“very unlike me”) to 4 (“very like me”). The Korean version of the IPSM comprises five subscales, namely, interpersonal awareness (four items, e.g., “I worry about the effect I have on other people”), need for approval (four items, e.g., “I will go out of my way to please someone I am close to”), separation anxiety (six items, e.g., “I feel insecure when I say goodbye to people”), timidity (five items, e.g., “I will do something I don’t want to do rather than offend or upset someone”), and fragile inner self (five items, e.g., “My value as a person depends enormously on what others think of me”).

#### Clinical Assessment of Psychopathology: Objective Symptoms

Psychopathology was assessed clinically using the Positive and Negative Syndrome Scale (PANSS), the Clinical Global Impression Schizophrenia (CGI-SCH), the Calgary Depression Scale for Schizophrenia (CDSS), and the Social and Occupational Functioning Assessment Scale (SOFAS). The PANSS (44, 53) uses interview and reports of family members to assess the severity of the two common symptom types in schizophrenia—positive and negative—as well as the general psychopathology of the patient. The CGI-SCH (54) consists of only two categories: severity of illness and degree of change. Each category contains five different ratings (positive, negative, depressive, cognitive, and global). The CDSS (55–57) was used to measure depressive symptoms of schizophrenia. The SOFAS (58) is a single-item scale used to indicate the individual’s level of social and occupational functioning across a continuum, ranging from a state of optimum functioning to a state of important functional impairment. It measures the level of social and occupational functioning, without taking symptoms into account.

### Magnetic Resonance Imaging and Voxel-Based Morphometry Analysis

Magnetic resonance imaging (MRI) was performed using a 3-T scanner (MAGNETOM Verio; Siemens, Erlangen, Germany). To minimize image distortion due to head motion, restraining foam pads were used. High-resolution, T1-weighted structural brain MR images were acquired using the following acquisition parameters: 256 × 246 acquisition matrix, 270 × 270 or 250 × 250 field-of-view, 0.527 × 0.527 × 1 or 0.488 × 0.488 × 1 voxel size, a total of 262,144 voxels, an echo time (TE) of 2.45 ms, a repetition time (TR) of 1900 ms, a 1-mm slice thickness, and a flip angle of 9°. Specifically, among the patients with FEP, 22 were imaged using a voxel size of 0.488 × 0.488 × 1, while 12 had a voxel size of 0.527 × 0.527 × 1). Among the HCs, 19 were imaged using a voxel size of 0.488 × 0.488 × 1), while 15 had a voxel size of 0.527 × 0.527 × 1. Therefore, we controlled for voxel size as covariate factor in the statistical analysis.

Voxel-based volumetry (VBM) was conducted using the Computational Anatomy Toolbox (CAT12; developed by Christian Gaser, University of Jena, http://dbm.neuro.uni-jena.de/cat), which is provided in the SPM12 software package (Wellcome Department of Cognitive Neurology, London, UK) (59, 60) and can be run using MATLAB R2016b (Mathworks Inc.). The structural T1 images were affine registered to an ICBM East Asian template and normalized using the DARTEL algorithm (61). The images were then segmented into gray matter, white matter, and cerebrospinal fluid (62). Jacobian-transformed tissue probability maps were used to modulate the images and estimate volume differences in gray matter. The brain volume was estimated in 148 regions of the Neuromorphometrics atlas, which is available in SPM12 (Neuromorphometrics Inc., http://neuromorphometrics.com). The frontal lobe, amygdala, hippocampus, insula, and STG were analyzed as regions of interest (ROIs) of brain volume, since these areas were associated with major pathology of first episode psychosis (63–69). Among 148 regions of the Neuromorphometrics atlas, 30 regions matched with the five ROIs were selected for analysis (Frontal lobe: L/R frontal operculum, L/R frontal pole, L/R medial frontal cortex, L/R middle frontal gyrus, L/R superior frontal gyrus medial segment, L/R opercular part of the inferior frontal gyrus, L/R orbital part of the inferior frontal gyrus, L/R superior frontal gyrus, L/R triangular part of the inferior frontal gyrus, amygdala: L/R Amygdala, hippocampus: L/R Hippocampus, L/R parahippocampal gyrus, insula: L/R anterior insula, L/R posterior insula, and STG: L/R superior temporal gyrus).

### Statistical Analysis

Independent t-tests were used to compare the demographic data. A multivariate analysis of covariance (MANCOVA) was used to compare psychological measures and brain volume between the patients with the FEP and the HC. A partial correlation analysis was performed to examine the relationship between psychological measures and brain volume which had differed significantly by two groups through the preceding MANCOVA analysis. Additional regression analysis using the SPSS Macro PROCESS for SPSS 2.16.3 (70) was performed to examine the moderation effect of the groups. Age, sex, years of education, antipsychotic dosage, status of medication use (taking drugs, drug naïve, drug free, HC), and duration of illness (DI) were considered as covariates. Antipsychotic dosage, status of medication use (taking drugs, drug naïve, drug free, and HC), and duration of illness (DI) were not considered as covariates during partial correlation for the HC group. When volume was included in the analysis, the total intracranial volume (TIV) and voxel size were considered as additional covariates to correct for different brain sizes (71). The significance level was set at *p* < 0.05 (two-tailed). For multiple correction, 5,000 times resampled bootstrapping method was applied for the MANCOVA, partial correlation, and moderated regression analyses (72). Statistical analyses were performed using SPSS 21 (SPSS, Inc., Chicago, IL, USA).

## Results

### Demographic and Psychological Characteristics

The patients with FEP did not differ in age and sex from the HCs, although the number of years of education differed significantly between the two groups (*p* < 0.001), so we controlled for years of education as a covariate in the statistical analysis. The demographic and psychological characteristics of the participants are shown in **Table 1**. Among the 34 FEP patients, eight were drug naive, six were drug free, and 20 were taking antipsychotic medication (risperidone: n = 3, olanzapine: n = 3, paliperidone: n = 4, aripiprazole: n = 6, blonanserin: n = 2, amisulpride: n = 1, paliperidone palmitate: n = 1) during the study.

**Table 1:** Comparison of demographic and clinical characteristics between first episode psychosis (FEP) patients and healthy control (HC) participants.

	FEP (n = 34)	HC (n = 34)	*t* or *χ²*	*p*
	*Mean (SD)* or N (%)			
**Age (years)**	28.353 (7.261)	31.647 (6.764)	-1.936	0.057
**Gender**			1.482	0.223
Male	16 (47.1%)	21 (61.8%)		
Female	18 (52.9%)	13 (38.2%)		
**Education (years)**	13.324 (2.041)	16.618 (2.349)	-6.173	**<0.001**
**DI (months)**	16.559 (20.534)			
**Dosage of antipsychotics (CPZ equivalent, mg)**	226.050 (552.931)			
**PANSS**				
Positive	17.206 (8.007)			
Negative	13.912 (5.605)			
General	31.412 (10.234)			
Total	62.529 (19.568)			
**CDSS**	7.176 (6.093)			
**CGI-SCH**				
Positive	4.000 (1.633)			
Negative	3.029 (1.337)			
Depressive	2.765 (1.793)			
Cognitive	2.353 (1.070)			
Overall	4.412 (1.305)			
**SOFAS**	58.235 (12.726)			

t, χ², and p values represent statistically significant differences between FEP and healthy participants. DI, duration of illness; CDSS, Calgary depression scale for schizophrenia; CGI-SCH, clinical global impression schizophrenia; PANSS, positive and negative syndrome scale; SOFAS, social and occupational functioning assessment scale. Bold texts present significant results or significant and larger values when comparing between groups.

### Self-Awareness of Psychopathology: Subjective Symptoms

MANCOVA analysis was applied to all self-awareness of psychopathology measures, including the six subscales of the PRMQ, the three subscales of the RRS, and the five subscales of the IPSM, to examine the differences between the two groups by generating 5,000 bootstrapped samples for multiple comparison (72). Age, sex, years of education, dosage of antipsychotics, status of medication use, and DI were controlled as covariates. There were significant differences between the two groups in the PRMQ, RRS, and IPSM. In the PRMQ, all six subscale scores were significantly higher in the FEP group than in the HC group: prospective (17.206 ± 6.777 vs. 13.941 ± 5.662, *F*(1, 59) = 10.108, *p* = 0.002), retrospective (16.324 ± 5.250 vs. 12.676 ± 4.511, *F*(1, 59) = 19.001, *p* < 0.001), short-term (16.882 ± 5.989 vs. 13.235 ± 4.912, *F*(1, 59) = 12.465, *p* = 0.001), long-term (16.647 ± 5.969 vs. 13.382 ± 5.176, *F*(1, 59) = 15.139, *p* < 0.001), self-cued (17.882 ± 6.158 vs. 13.588 ± 5.286, *F*(1, 59) = 13.705, *p* < 0.001), environmentally-cued (15.647 ± 6.075 vs. 13.029 ± 4.796, *F*(1, 59) = 12.982, *p* = 0.001), and total memory impairment self-assessment score (33.529 ± 11.471 vs. 26.618 ± 9.912, *F*(1, 59) = 14.807, *p* < 0.001). In the RRS, the depression-related rumination scale (26.353 ± 8.790 vs. 21.000 ± 6.325, *F*(1, 59) = 9.508, *p* = 0.003) and total ruminative response scale (47.294 ± 15.712 vs. 39.353 ± 11.484, *F*(1, 59) = 6.153, *p* = 0.016) were significantly higher in the FEP group than in the HC group. In the IPSM, interpersonal awareness (9.618 ± 3.420 vs. 8.294 ± 2.529, *F*(1, 59) = 4.181, *p* = 0.045), separation anxiety (11.647 ± 4.206 vs. 9.294 ± 3.010, *F*(1, 59) = 5.185, *p* = 0.026), and fragile inner self (9.353 ± 3.757 vs. 7.294 ± 2.493, *F*(1, 59) = 7.998, *p* = 0.006) were significantly higher in the FEP group than in the HC group. These results are presented in **Table 2**.

**Table 2:** Comparison of scores on subjective cognitive assessment scales between first episode psychosis (FEP) patients and healthy control (HC) participants.

	FEP (n = 34)	HC (n = 34)	*p* †
	*Mean* (*SD*)		
**Prospective retrospective memory questionnaire (PRMQ)**			
Prospective	**17.206 (6.777)**	13.941 (5.662)	0.002**
Retrospective	**16.324 (5.250)**	12.676 (4.511)	<0.001***
Short-term	**16.882 (5.989)**	13.235 (4.912)	0.001**
Long-term	**16.647 (5.969)**	13.382 (5.176)	<0.001***
Self-cued	**17.882 (6.158)**	13.588 (5.286)	<0.001***
Environmentally-cued	**15.647 (6.075)**	13.029 (4.796)	0.001**
Total	**33.529 (11.471)**	26.618 (9.912)	<0.001***
**Ruminative response scale (RRS)**			
Depression related	**26.353 (8.790)**	21.000 (6.325)	0.003**
Reflection	9.353 (3.884)	9.353 (2.953)	0.185
Brooding	11.588 (3.948)	9.000 (3.153)	0.087
Total	**47.294 (15.712)**	39.353 (11.484)	0.016*
**Interpersonal sensitivity measure (IPSM)**			
Interpersonal awareness	**9.618 (3.420)**	8.294 (2.529)	0.045*
Need for approval	9.324 (2.495)	9.412 (2.244)	0.272
Separation anxiety	**11.647 (4.206)**	9.294 (3.010)	0.026*
Timidity	10.676 (3.699)	10.000 (3.238)	0.599
Fragile inner self	**9.353 (3.757)**	7.294 (2.493)	0.006**
Total	50.676 (14.869)	44.294 (11.930)	0.177

^†^Bootstrapping with 5,000 replications.

*p < 0.05, **p < 0.01, ***p < 0.001. Bold texts present significant results or significant and larger values when comparing between groups.

### Brain Volume

MANCOVA analysis was applied to brain volume in 30 subregions, including the five ROIs that are associated with major pathology in FEP (frontal lobe, amygdala, hippocampus, insula, and STG) (63–69), to examine the differences between the two groups by generating 5,000 bootstrapped samples for multiple comparison (72). Age, sex, years of education, antipsychotic dosage, status of medication use, DI, TIV, and voxel size were controlled as covariates. There were significant volume differences between the two groups in the amygdala, hippocampus, and STG. The volumes of the left amygdala (0.845 ± 0.111 vs. 0.931 ± 0.085, *F*(1, 58) = 7.001, *p* = 0.010), right amygdala (0.825 ± 0.106 vs. 0.914 ± 0.085, *F*(1, 58) = 5.520, *p* = 0.022), left hippocampus (3.145 ± 0.352 vs. 3.404 ± 0.341, *F*(1, 58) = 8.559, *p* = 0.005), right hippocampus (3.494 ± 0.350 vs. 3.763 ± 0.310, *F*(1, 58) = 5.515, *p* = 0.022), left parahippocampal gyrus (2.920 ± 0.282 vs. 3.182 ± 0.304, *F*(1, 58) = 5.106, *p* = 0.028), left STG (6.241 ± 0.783 vs. 6.821 ± 0.890, *F*(1, 58) = 6.161, *p* = 0.016), and right STG (rSTG; 6.618 ± 0.777 vs. 7.255 ± 0.899, *F*(1, 58) = 4.146, *p* = 0.046) were significantly lower in the FEP group than in the HC group. These results are presented in **Table 3**.

**Table 3:** Comparison of brain volume between first episode psychosis (FEP) patients and healthy control (HC) participants.

	FEP (n = 34)	HC (n = 34)	*p* †
	*Mean* (*SD*)		
**Frontal lobe**			
Left frontal operculum	1.918 (0.265)	2.069 (0.317)	0.303
Right frontal operculum	2.009 (0.305)	2.191 (0.315)	0.969
Left frontal pole	2.802 (0.431)	2.948 (0.327)	0.994
Right frontal pole	3.270 (0.443)	3.501 (0.428)	0.907
Left medial frontal cortex	1.702 (0.332)	1.890 (0.247)	0.709
Right medial frontal cortex	1.858 (0.297)	2.070 (0.309)	0.827
Left middle frontal gyrus	17.957 (2.184)	19.470 (2.444)	0.187
Right middle frontal gyrus	17.821 (2.386)	19.067 (2.399)	0.322
Left superior frontal gyrus medial segment	5.803 (0.770)	6.288 (0.807)	0.753
Right superior frontal gyrus medial segment	7.087 (1.045)	7.719 (0.952)	0.843
Left opercular part of the inferior frontal gyrus	3.244 (0.421)	3.517 (0.601)	0.494
Right opercular part of the inferior frontal gyrus	3.418 (0.515)	3.701 (0.529)	0.684
Left orbital part of the inferior frontal gyrus	1.358 (0.229)	1.401 (0.195)	0.143
Right orbital part of the inferior frontal gyrus	1.394 (0.239)	1.432 (0.221)	0.182
Left superior frontal gyrus	13.306 (1.731)	14.116 (1.577)	0.722
Right superior frontal gyrus	13.349 (1.688)	14.378 (1.907)	0.820
Left triangular part of the inferior frontal gyrus	2.987 (0.427)	3.222 (0.506)	0.473
Right triangular part of the inferior frontal gyrus	3.146 (0.480)	3.372 (0.487)	0.823
**Amygdala**			
Left Amygdala	0.845 (0.111)	**0.931 (0.085)**	0.010*
Right Amygdala	0.825 (0.106)	**0.914 (0.085)**	0.022*
**Hippocampus**			
Left Hippocampus	3.145 (0.352)	**3.404 (0.341)**	0.005**
Right Hippocampus	3.494 (0.350)	**3.763 (0.310)**	0.022*
Left parahippocampal gyrus	2.920 (0.282)	**3.182 (0.304)**	0.028*
Right parahippocampal gyrus	2.867 (0.295)	3.076 (0.334)	0.329
**Insula**			
Left anterior insula	4.624 (0.474)	4.769 (0.494)	0.332
Right anterior insula	4.680 (0.595)	4.896 (0.495)	0.279
Left posterior insula	2.253 (0.252)	2.363 (0.261)	0.482
Right posterior insula	2.616 (0.281)	2.743 (0.278)	0.169
**Superior temporal gyrus**			
Left superior temporal gyrus	6.241 (0.783)	**6.821 (0.890)**	0.016*
Right superior temporal gyrus	6.618 (0.777)	**7.255 (0.899)**	0.046*

^†^Bootstrapping with 5,000 replications. *p < 0.05, **p < 0.01. Bold texts present significant results or significant and larger values when comparing between groups.

### Correlation Analyses of Psychological Measures and Brain Volume

Partial correlation analysis was applied to the psychological measures and to the volume of the left/right amygdala, left/right hippocampus, left hippocampal gyrus, and left/right STG, all of which had differed significantly between the two groups in the preceding MANCOVA analysis, to examine their correlation with psychological symptoms by generating 5,000 bootstrapped samples for multiple comparison (72). Age, sex, years of education, TIV, and voxel size were controlled as covariates in all participants. Antipsychotic dosage, status of medication use, and DI were controlled in FEP patients.

Among all participants, need for approval (IPSM) was significantly associated with the left amygdala (*r* = 0.284, *p* = 0.028) and right hippocampus (*r* = 0.289, *p* = 0.025). The left STG was correlated with the PRMQ (prospective: *r* = -0.255, *p* = 0.050; retrospective: *r* = -0.310, *p* = 0.016; short-term: *r* = -0.259, *p* = 0.046; long-term: *r* = -0.303, *p* = 0.019; self-cued: *r* = -0.316, *p* = 0.014; total PRMQ: *r* = -0.290, *p* = 0.025) and with the RRS (depression related: *r* = -0.355, *p* = 0.005; reflection: *r* = -0.344, *p* = 0.007; brooding: *r* = -0.340, *p* = 0.008; total RRS: *r* = -0.370, *p* = 0.004).

In the FEP group, the volume of the left hippocampus was significantly correlated with the CDSS (*r* = 0.480, *p* = 0.013). The right hippocampus was related to the need for approval (IPSM subscale) (*r* = 0.388, *p* = 0.050), CDSS (*r* = 0.543, *p* = 0.004), and depressive CGI-SCH (*r* = 0.460, *p* = 0.018). All correlation results between clinical assessment and brain volume are presented in **Table 4**. The right STG volume was associated with the PRMQ (prospective: *r* = 0.393, *p* = 0.047; short-term: *r* = 0.419, *p* = 0.033; environmentally-cued: *r* = 0.494, *p* = 0.010; total PRMQ: *r* = 0.414, *p* = 0.036).

**Table 4:** Correlation between brain volume and clinical assessment of psychopathology in first episode psychosis (FEP) patients.

	*r* with left Amygdala	*r* with right Amygdala	*r* with left Hippocampus	*r* with right Hippocampus	*r* with left parahippocampal gyrus	*r* with left superior temporal gyrus	*r* with right superior temporal gyrus
**PANSS**							
Positive	0.188	0.064	0.278	0.360	0.352	-0.003	-0.037
Negative	0.138	0.047	0.112	0.073	0.012	-0.132	-0.282
General	0.258	0.094	0.345	0.393*	0.335	-0.014	-0.213
Total	0.254	0.090	0.327	0.373	0.320	-0.049	-0.215
**CDSS**	0.293	0.276	0.480*	0.543**	0.093	0.091	0.081
**CGI-SCH**							
Positive	0.137	-0.110	0.140	0.329	0.112	-0.130	0.079
Negative	0.108	-0.071	0.026	-0.014	-0.033	-0.127	-0.133
Depressive	0.130	-0.056	0.215	0.460*	0.169	-0.027	0.112
Cognitive	-0.101	-0.144	0.009	-0.071	0.069	-0.169	0.006
Overall	0.216	-0.025	0.116	0.371	0.051	0.015	0.227
**SOFAS**	-0.130	0.066	-0.217	-0.183	-0.192	0.294	0.389

*p < 0.05, **p < 0.01, bootstrapping with 5,000 replications was used. Bold texts present significant results or significant and larger values when comparing between groups.

In the HC group, the left STG volume was negatively correlated with the PRMQ (retrospective: *r* = -0.389, *p* = 0.037) and the RRS (depression related: *r* = -0.466, *p* = 0.011; reflection: *r* = -0.432, *p* = 0.019; brooding: *r* = -0.526, *p* = 0.003; total RRS: *r* = -0.507, *p* = 0.005). The rSTG volume was negatively related to the PRMQ (prospective: *r* = -0.423, *p* = 0.022; retrospective: *r* = -0.446, *p* = 0.015; short-term: *r* = -0.486, *p* = 0.007; long-term: *r* = -0.388, *p* = 0.038; self-cued: *r* = -0.401, *p* = 0.031; environmentally-cued: *r* = -0.475, *p* = 0.009; total PRMQ: *r* = -0.443, *p* = 0.016), the RRS (depression related: *r* = -0.431, *p* = 0.020; total RRS: *r* = -0.420, *p* = 0.023), and the IPSM (timidity: *r* = -0.406, *p* = 0.029).

No other pairs showed any significant correlation between brain volume of ROIs and psychological measures.

### Moderated Regression Analysis of Self-Awareness of Psychopathology and rSTG Volume

From the previous correlation analysis comparing five ROIs (frontal lobe, amygdala, hippocampus, insula, and STG) with self-awareness of psychopathology, only rSTG volume showed a noticeable difference between the FEP and HC groups. To examine the interaction between group and self-awareness of psychopathology (the PRMQ, RRS, and IPSM) on rSTG volume, moderation analyses were used. Each self-awareness scale was set as an independent variable, while rSTG volume was set as the dependent variable and the group (FEP vs. HC) was set as a moderator. The following variables were controlled for as covariates: age, sex, years of education, antipsychotic dosage, status of medication use, DI, TIV, and voxel size.

When the PRMQ score was set as the independent variable, the moderation model was significant (*R²* = 0.656, *p* < 0.001), as was the moderation effect, since the *R²* of the interaction was higher (△*R²* = 0.074, △*F* = 12.074, *p* = 0.001). The coefficient of the PRMQ total (*B* = -0.028, *p* = 0.009), the coefficient of group (*B* = -2.703, *p* < 0.001), and the interaction between PRMQ total and group (*B* = 0.049, *p* = 0.001) were significant. The HC group showed a significant negative effect (Effect = -0.028, *p* = 0.009), while the FEP group showed a significant positive effect (Effect = 0.021, *p* = 0.048).

When RRS score was set as the independent variable, the moderation model was significant (*R²* = 0.624, *p* < 0.001), as was the moderation effect was significant, since the *R²* of the interaction was higher (△*R²* = 0.034, △*F* = 5.101, *p* = 0.028). The coefficient of the RRS total (*B* = -0.024, *p* = 0.016), the coefficient of group (*B* = -2.206, *p* = 0.007), and the interaction between RRS total and group (*B* = 0.027, *p* = 0.028) were significant. The HC group showed a significant negative effect (Effect = -0.024, *p* = 0.016), while the FEP group showed an insignificant positive effect (Effect = 0.004, *p* = 0.597).

When the IPSM score was set as the independent variable, the moderation model was significant (*R²* = 0.615, *p* < 0.001), as was the moderation effect was significant, since the *R²* of the interaction was higher (△*R²* = 0.032, △*F* = 4.608, *p* = 0.036). Although the coefficient of the IPSM total (*B* = -0.013, *p* = 0.181) was not significant, the coefficient of group (*B* = -2.523, *p* = 0.004) and the interaction between IPSM total and group (*B* = 0.027, *p* = 0.036) were significant. The HC group showed an insignificant negative effect (Effect = -0.013, *p* = 0.181), while the FEP group showed an insignificant marginally significant positive effect (Effect = 0.014, *p* = 0.067).

**Figure 1:**
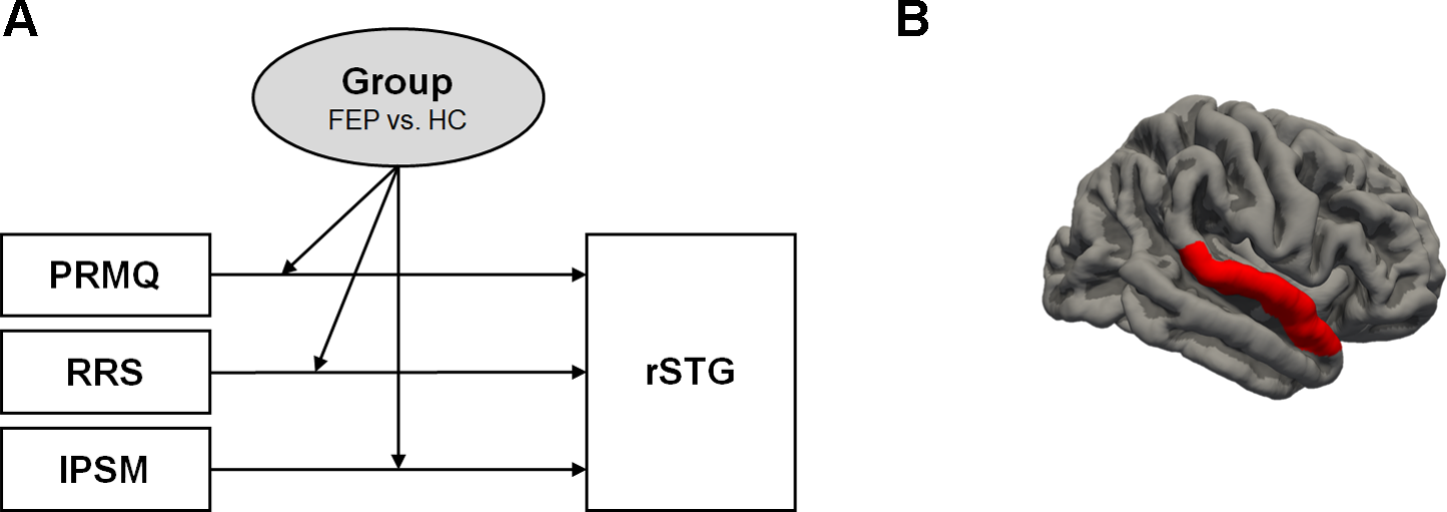
**(A)** Moderation of the effect of self-reported psychopathology evaluations (prospective and retrospective memory questionnaire (PRMQ), ruminative response scale (RRS), and interpersonal sensitivity measure (IPSM)) on right superior temporal gyrus (rSTG) volume at values of the moderator group. **(B)** rSTG region (red colored).

**Figure 2:**
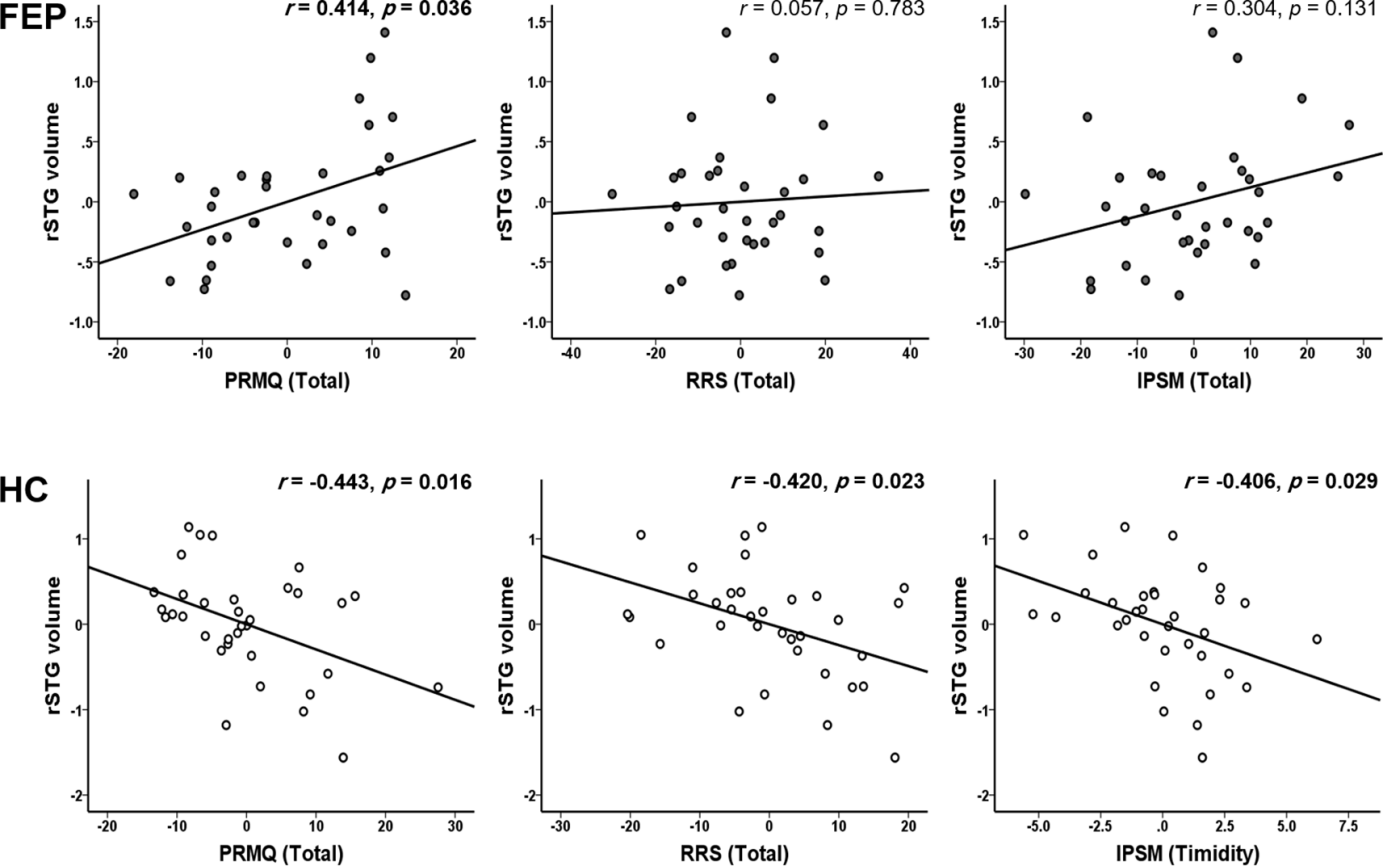
Significant group dependent moderation effect between self-reported psychopathology evaluations (prospective and retrospective memory questionnaire (PRMQ), ruminative response scale (RRS), and interpersonal sensitivity measure (IPSM)) and right superior temporal gyrus (rSTG) volume. In first episode psychosis (FEP) group, the PRMQ score significantly positively correlated with the rSTG volume, also RRS and IPSM, positively correlated with the rSTG volume. In healthy control (HC) group, the PRMQ, RRS, and IPSM scores significantly negatively correlated with the rSTG volume.

**Figure 1** presents the moderation effects of the PRMQ, RRS, and IPSM on the rSTG volume in each moderator group. **Figure 2** shows the opposite direction of the effect in each group. In FEP group, the PRMQ, RRS, and IPSM scores positively correlated with the rSTG volume. In HC group, the scores negatively correlated with the rSTG volume.

## Discussion

The present study used the PRMQ, RRS, and IPSM to explore brain volume and self-awareness of psychopathology in patients with FEP. In the FEP group, the volume of the amygdala, hippocampus, and STG were significantly lower than in the HC group, while the PRMQ, RRS, and IPSM scores were significantly higher. Significant group-dependent moderation effects were found between self-awareness of psychopathology (PRMQ, RRS, and IPSM scores) and rSTG volume. The PRMQ score was positively associated with rSTG volume in the FEP group. However, the relationship was negative in the HC group.

In patients with FEP, the amygdala, hippocampus, and STG showed significantly lower volumes than in HCs. Reduced amygdala volume has repeatedly been demonstrated among patients with chronic schizophrenia (73–77), although another study reported no such volume reduction (78). Several studies have found that amygdala volume in patients with early-stage schizophrenia is smaller than in HCs (79, 68), while others have found no significant difference (80, 81). Rich et al. (82) demonstrated smaller amygdala volume during early illness than during chronic-stage schizophrenia. Meta-analyses have confirmed reduced hippocampal volume in patients with schizophrenia (83, 84), and some studies have identified decreased hippocampal volume in patients with chronic schizophrenia (85, 86), as well as in those with early schizophrenia (85, 84). The hippocampus plays a role in cognitive function, particularly memory (87). In schizophrenia, the hippocampus and parahippocampus have been correlated with accuracy and performance speed, memory, and executive function and abstraction (88, 89).

Smaller STG volume occurs in patients with schizophrenia when compared with HCs (90–93, 36, 94, 95). This volumetric reduction in the STG is progressive over time in individuals at ultra-high risk for psychosis, as well as in those with childhood onset and in those with schizophrenia and FEP (96, 97, 94, 95). In addition, a right-side dominant STG volumetric reduction has often been reported in both first-episode (98, 99) and chronic schizophrenia (76, 100). The STG contains the “social brain” network (101, 102, 103) and is responsible for auditory processing, language functions, and auditory memory (104, 105).

In the present study, subjective self-report scales were used to measure psychopathology. Although self-reporting tools have consistently demonstrated high reliability, meta-memory (i.e., individuals’ beliefs about their own memory ability) is not always highly correlated with actual performance in objective memory tests or clinical observation (106, 107). The present results showed that higher self-awareness of psychopathology scores positively associated with rSTG volume in the FEP group (PRMQ was significant, RRS and IPSM were insignificant). However, these correlations were negative in the HC group. This correlation was especially robust in the PRMQ. These results suggest that rSTG volume has significant implications for self-reporting memory. Since the PRMQ assesses a patient’s own amnesia experience, high scores imply high awareness of memory impairment (108). In addition, PRMQ is used to measure cognitive functions, as well as the broader neurobehavioral changes that are likely to occur alongside compromised functioning, such as loss of insight (109–111).

Significant group-dependent moderation effects were found between self-awareness of psychopathology (PRMQ, RRS, and IPSM scores) and rSTG volume, positive effect in the FEP group, and negative effect in the HC group. In patients with schizophrenia, poor insight (lower self-awareness of psychopathology) is associated with reduced total brain volume (112, 113), ventricular enlargement (114), frontal lobe atrophy (115), reduced frontal lobe volume (116–118), and gray matter deficits in the cingulate gyrus (119, 118), temporal lobe (119, 120), parietal lobe (120), and precuneus (120). In addition to these findings, the results of our study could provide further evidence on the characteristics of an individual’s poor insight into his or her psychopathology.

Meanwhile, healthy older adults with subjective memory impairments, defined as the feeling of worsening memory with normal memory performance, show smaller brain structures (121–125), especially in the medial temporal lobe region (122–126), hippocampus (127, 128, 121, 129), and amygdala (122, 130, 131, 124, 132). Additionally, smaller gray matter volume is associated with excessive rumination in healthy adults (133–136). No previous studies have addressed the correlation between interpersonal sensitivity and brain volume. These previous findings suggest that self-awareness of psychopathology would reduce brain volume in healthy individuals.

This study had some limitations. Firstly, we did not measure objective cognitive impairment. Secondly, we could not measure brain MRI in a unified way. Instead, images were acquired in two voxel sizes, even though voxel size has been identified as a covariate to correct for different brain sizes (71).

In conclusion, we found different correlation patterns between brain volume and self-awareness of psychopathology in the FEP and HC groups. In the FEP group, self-awareness of psychopathology was associated with increased rSTG volume. However, the HC group showed decreased rSTG volume when they were aware their discomfort. Our results indicate that self-awareness of psychopathy impacts rSTG volume differently in patients with FEP and HCs.

## Data Availability Statement

The datasets for this manuscript are not publicly available because participants and guardians have not given consent for data sharing. Requests to access the datasets should be directed to SL (lshpss@hanmail.net).

## Ethics Statement

The studies involving human participants were reviewed and approved by the Ethics Committee of Chonbuk National University Hospital, Republic of Korea. The patients/participants provided their written informed consent to participate in this study.

## Author Contributions

J-YK contributed to analyzing data and wrote the paper. HJ collected the data and analyzed data. AK wrote sections of the manuscript. MJ wrote sections of the manuscript. S-HL and Y-CC supervised the study process and manuscript writing. All authors contributed to manuscript revision and read and approved the submitted version.

## Funding

This study was supported by a grant from the Korea Science and Engineering Foundation (KOSEF), funded by the Korean government (NRF-2018R1A2A2A05018505), a grant from the Korean Mental Health Technology R&D Project, Ministry of Health & Welfare, Republic of Korea (HM14C2608), and a grant of the Korea Health Technology R&D Project through the Korea Health Industry Development Institute (KHIDI) and the Ministry of Health & Welfare, Republic of Korea (grant number HI18C2383).

## Conflict of Interest

The authors declare that the research was conducted in the absence of any commercial or financial relationships that could be construed as a potential conflict of interest.
